# Rationale and design of the SMILe (Spinal Morphine or Intravenous Lidocaine) study: protocol for a multicentre randomised clinical trial in southern Sweden on whether spinal morphine improves postoperative recovery compared to intravenous lidocaine in patients undergoing robot-assisted upper urinary tract surgery

**DOI:** 10.1136/bmjopen-2025-113402

**Published:** 2026-05-06

**Authors:** Martin Holmberg, Michelle Chew, Lena Nilsson, Per Skoglund, Hans Bahlmann

**Affiliations:** 1 Department of Biomedical and Clinical Sciences, Linköping University, Linköping, Östergötland County, Sweden; 2 Department of Anaesthesiology and Intensive Care in Linköping, Linköping University, Linköping, Sweden; 3 Department of Urology in Östergötland, Linköping University, Linköping, Sweden

**Keywords:** Anaesthesia in urology, CLINICAL PHYSIOLOGY, Hemodynamics, Patient Reported Outcome Measures, PAIN MANAGEMENT, Randomized Controlled Trial

## Abstract

**Introduction:**

Robot-assisted laparoscopic surgical procedures are commonly perceived to result in fast recovery; however, the postoperative course can be challenging for many patients. We have previously found severe pain and a significant decrease in the patient-reported outcome measure Quality-of-Recovery 15 (QoR-15) in a cohort of patients undergoing robot-assisted upper urinary tract surgery. In similar settings, intrathecal analgesia is sometimes used to improve recovery; however, its benefits have not been established. Therefore, this study aims to examine the effects of intrathecal analgesia in this setting compared with an active comparator intravenous lidocaine.

**Methods and analysis:**

In this randomised, assessor-blinded multicentre trial, 220 patients scheduled for robot-assisted upper urinary tract surgery under general anaesthesia are recruited after obtaining informed consent. They are randomised to receive either intrathecal analgesia or an intraoperative infusion with lidocaine. The primary study outcome is the decrease in QoR-15 from baseline to postoperative day 1. Other outcomes of interest include postoperative pain, length of stay and postoperative complications. Differences in intraoperative haemodynamics and postoperative inflammatory parameters will also be analysed.

**Ethics and dissemination:**

This study has been approved by the Swedish Medical Products Agency (5.1.1-2023-69740 and 5.1.2-2025-030145). The results of this study will be presented at national and international meetings and submitted for publication in peer-reviewed international medical journals.

**Trial registration number:**

NCT06349668.

**STRENGTHS AND LIMITATIONS OF THIS STUDY**

- Multicentre design increases external validity.
- Use of active comparator increases clinical benefit of key results.
- Recovery is measured in multiple ways at multiple time points, as are effects on haemodynamic and inflammatory parameters.
- Double-blind set-up not possible.
- Absence of control group with no treatment.

## Introduction

 Robot-assisted laparoscopic surgery is associated with a reduction in surgical trauma compared with laparotomy. This leads to less bleeding and faster patient recovery.^1^ However, patients who undergo laparoscopic surgery may experience more pain during the first day than patients who have undergone the same procedure with open technique.^2^ In our own material, we have noted severe postoperative pain in a majority of patients who underwent robot-assisted laparoscopic upper urinary tract surgery (unpublished data).

In the case of major open abdominal surgery, in addition to general anaesthesia, epidural analgesia is considered standard of care to enable adequate postoperative pain relief.^3^ In laparoscopic surgery, however, the benefit of epidural analgesia is limited.^4 5^ Spinal (intrathecal) analgesia could be an option in laparoscopic surgery.

In abdominal surgery, spinal analgesia is most commonly applied using two components. Bupivacaine, a local anaesthetic, has a relatively short (2–6 hours) duration of action after administration and is effective mainly during surgery, and morphine, which has a longer duration of action and provides pain relief up to 24 hours postoperatively. This mixture will henceforth be referred to as ‘morphine spinal’.

Morphine spinal has several disadvantages: it must (in many countries) be administered by an anaesthesiologist and can delay the start of surgery, it can cause perioperative hypotension as well as postoperative nausea and troublesome itching. In very rare cases, the method can lead to severe neurological complications caused by local bleeding or infection.^6^ Respiratory depression also is a known complication of morphine spinal.^7^ These findings suggest that an alternative pain relief strategy that is as effective as morphine spinal but lacks its disadvantages would be preferable.

Potential candidates for this alternative strategy include drugs such as non-steroidal anti-inflammatory drugs (NSAIDs), cyclooxygenase (COX)-2 inhibitors, clonidine, lidocaine, ketamine and magnesium.^8 9^ However, their effectiveness has not been definitively established,^10^,^12^ some preparations are not available in Sweden (nefopam and metamizole) and there is also uncertainty about possible negative effects related to kidney function (NSAIDs and COX-2 inhibitors) and malignancy (ketamine, clonidine and magnesium).^13^,^20^ This is of great importance in patients undergoing upper urinary tract surgery since they are often affected by malignant kidney disease.

The only alternative adjunctive treatment that is considered to have the possibility of approaching an equivalent effect to morphine spinal and that is not associated with renal deterioration or a negative effect on tumour progression is intravenous administration of lidocaine.

Lidocaine is a local anaesthetic that has been used since the 1950s. Lidocaine can, among other routes, be given intravenously, epidurally and as infiltration anaesthesia.^21^ When administered intravenously, lidocaine can reduce pain, counteract hyperalgesia and dampen the inflammatory response during surgery,^22 23^ and is thus a possible strategy to improve patient recovery after abdominal surgery.^24 25^ A meta-analysis examining the effect of intravenous lidocaine in laparoscopic surgery showed that lidocaine resulted in a reduced need for opioids, less pain and less postoperative nausea.^26^ However, a Cochrane analysis from 2018 examining the effect of intravenous lidocaine in all types of surgery showed only a small effect on pain early (0–4 hours) postoperatively.^10^

The evidence for the benefits of intravenous lidocaine in urological laparoscopic surgery is sparse. In open kidney surgery, there was less need for opioids, less pain during movement and less postoperative nausea.^27^ A three-arm study comparing intravenous lidocaine with a spinal anaesthetic (with the local anaesthetic bupivacaine and the opioid fentanyl) or a so-called TAP (transabdominal plane) blockade in prostatectomies (both open and robot-assisted) showed no difference in postoperative recovery.^28^ Lauwick *et al.* reported that patients receiving intravenous lidocaine required less opioids and performed better on a functional walking test the day after a robot-assisted prostatectomy.^29^ In laparoscopic renal surgery, a randomised trial showed no improvement with lidocaine infusion in terms of pain, opioid requirement or length of stay.^11^ In contrast, a retrospective observational study showed a reduction in opioid requirement, less pain and faster recovery of bowel function after the introduction of intravenous lidocaine in patients who underwent laparoscopic nephrectomy.^30^

The benefit of combining general anaesthesia with morphine spinal is expected to be primarily visible through an effect on so-called ‘patient-reported outcome measures’ (PROM). The importance of using these patient-centred outcome measures when trying to improve perioperative care has recently been described.^31^ Quality-of-Recovery 15 (QoR-15), one of the several tests based on PROM, is often recommended as a recovery outcome measure in clinical trials.^32 33^

The QoR-15 measures patient recovery after surgery in the five domains of pain, physical well-being, physical independence, psychological support and emotional status. The QoR-15 provides a more complete picture of patient recovery compared with, for example, focusing solely on pain, and can be used to measure the quality of perioperative care.^34^ A change of 6–8 points has been defined as the minimal clinically important difference (MCID).^35 36^ Alternatively, a QoR-15 value of 118 has been indicated as the lowest value associated with an acceptable level of postoperative recovery (patient-accepted symptom state).^35^

In addition to providing pain relief, a morphine spinal also blocks the sympathetic nervous system, which through arterial and venous vasodilatation frequently results in low blood pressure. On the other hand, robot-assisted laparoscopic surgery itself stimulates the sympathetic nervous system, which results in increased blood pressure.^37^ It has not been studied how low blood pressure should be treated in a patient with a morphine spinal undergoing robot-assisted laparoscopic surgery. More knowledge about this enables a physiologically correct treatment, which is important especially when caring for patients with cardiovascular diseases who undergo robot-assisted laparoscopic surgery.

The surgical procedure is a critical event for cancer patients, as small parts of the tumour might spread into the bloodstream. A disturbed immune system, due to, among other factors, the inflammatory reaction caused by the surgical trauma and pain, can increase the risk of these small tumour parts developing into metastases.^38^ In surgery for benign conditions, a strong inflammatory response can have a negative impact on wound healing and increase the risk of complications. The effect of morphine spinal on the sympathetic nervous system affects the inflammatory response of the body to surgery, which in turn can be of importance to the risk of recurrence in cancer surgery.^39^ Morphine also has direct effects on the immune system, which can affect the recovery and recurrence rate of cancer.^40^ It is therefore of interest to compare different inflammation markers in patients with or without morphine spinal.

We therefore aim to compare the effects of a morphine spinal on recovery and well-being with intraoperative lidocaine infusion in patients undergoing robot-assisted upper urinary tract surgery. In a subgroup, the haemodynamic and inflammatory effects of both treatments are studied.

## Methods and analysis

### Study design, setting and population

This is a randomised prospective phase III (therapeutic confirmatory) multicentre study. The trial population consists of patients undergoing robot-assisted laparoscopic upper urinary tract surgery. The trial will start at the University Hospital in Linköping, Sweden. After the start of the study, other Swedish hospitals will be invited to participate in the trial.

Inclusion and exclusion criteria for participation in the trial are shown in table 1.

**Table 1:** Inclusion and exclusion criteria

Inclusion criteria	Exclusion criteria
The patient is listed for planned robot-assisted laparoscopic surgery on kidney and/or ureter at one of the hospitals participating in the trial The patient agrees to participate in the trial by signing ‘Informed consent’ after having received written and oral information	Anaesthetic risk classification (ASA) ≥4 Patients who are minors or declared incompetent, who have a severe psychiatric illness or cannot be expected to be able to understand the written and oral trial information due to severely impaired vision, hearing, cognition, reading ability or insufficient knowledge of Swedish Female patients who are pregnant or breastfeeding Premenopausal female patients who have not undergone sterilisation, hysterectomy, bilateral salpingectomy and/or bilateral oophorectomy, and who are not using highly effective contraception with low user dependence, and where there is no negative pregnancy test Emergency procedure Research staff is not available Extensive surgery on another organ during the same procedure The responsible anaesthesiologist recommends spinal or epidural analgesia Contraindications to morphine spinal, such as coagulopathy, previous back surgery with osteosynthesis material or severe aortic valve stenosis, or there is reason to expect a difficult puncture, such as pronounced scoliosis or pronounced obesity Contraindication to lidocaine infusion: proven allergy to local anaesthetics, renal failure with eGFR <30, liver failure due to acute hepatitis or cirrhosis corresponding to Child-Pugh ≥B, severe arrhythmia or heart failure problems (corresponding to NYHA IIIb or higher) and myasthenia gravis Previous participation in the trial

ASA, American Society of Anesthesiologists; eGFR, estimated glomerular filtration rate; NYHA, New York Heart Association.

### Patient and public involvement

This study was launched based on the finding of significant postoperative pain and discomfort reported by patients undergoing robot-assisted laparoscopic upper urinary tract surgery. The primary outcome of the study is based on the QoR-15 score, a PROM. Routines for patient recruitment and data collection have been modified to minimise patient discomfort related to the trial. Otherwise, patients have not been directly involved in the design and will not be directly involved in the recruitment to and conduct of the trial. Patients who require so will be informed of the results of the study, otherwise they are not involved in planning for the distribution of study results.

### Recruitment and consent

At the anaesthesia visit before surgery or at another time before surgery, the anaesthesiologist examines whether the potential subject meets the inclusion criteria and has no exclusion criteria. Potential trial subjects receive oral and written information about the trial and are asked after a period of consideration for participation. This includes information on data use and the handling of blood samples. Consent to participate in the trial is given verbally as well as in writing and is obtained by an anaesthesiologist.

### Inclusion and randomisation

Test subjects are included consecutively. Randomisation of the participating subjects takes place after inclusion in the trial, before the start of anaesthesia. For each participating hospital (apart from US Linköping, see the next section), Forum Östergötland prepares before the start of the trial 100 sequentially numbered, opaque and sealed envelopes in series of 20 with both groups distributed 1:1. For US Linköping, 200 envelopes are prepared in the same way. Forum Östergötland retains the randomisation codes.

### Interventions and measurements

Baseline values for QoR-15 and pain Numeric Rating Scale (NRS) are collected before the day of surgery. On the day of surgery, preoperative preparations on the ward follow local routines. A standardised premedication with acetaminophen and ondansetron is recommended. On arrival in the operating room all subjects are provided with basal monitoring including Bispectral Index (BIS). Induction and maintenance of anaesthesia is performed primarily with propofol and remifentanil or sevoflurane and fentanyl. Intubation and ventilator settings are managed according to local routine. Propofol or sevoflurane administration is controlled primarily by BIS, while the administration of remifentanil or other opioids intraoperatively is governed primarily by subject haemodynamics.

Haemodynamic treatment with fluids and drugs is performed according to local routine. A dose of 0.07 mg/kg actual body weight (ABW) intravenous morphine is recommended 30–60 min before the planned awakening, as well as 1 mcg/kg ABW intravenous fentanyl at the termination of remifentanil infusion when used.

Subjects randomised to the intervention group receive preoperative spinal analgesia with 15±5 mg bupivacaine and 0.2–0.3 mg conservative-free morphine using a 24G or smaller spinal pencil-point needle. In case of difficult puncture and/or pronounced discomfort, the procedure is aborted, and the subject is treated with intravenous lidocaine similar to the control group.

In subjects randomised to the control group, an intravenous infusion of lidocaine is started as soon as possible after induction. A bolus dose of 2 mg/kg ideal body weight (IBW, corresponding to a body mass index (BMI) of 22 kg/m^2^) is given over 10 minutes. An infusion of 2 mg/kg/hour IBW is then started. In patients with a BMI <22, ABW is used for calculation of bolus and maintenance doses. The infusion is terminated at the end of surgery or earlier in the event of unexplained severe hypotension or severe arrhythmias.

Additional intraoperative use of local anaesthetics, for example, for infiltration of trocar ports, is not allowed, with the exception of wound infiltration in case of unplanned conversion to laparotomy in patients in the morphine spinal group. Paracetamol 1 g is administered intravenously or orally every 6 hours to all patients. The addition of other adjuvant analgesics, such as NSAIDs, gabapentin, COX-2 inhibitors, clonidine and magnesium, is not permitted intraoperatively. Postoperatively, opiates and adjuvant analgesics are administered at the discretion of the responsible nurse or physician according to local routines. This includes the use of patient-controlled analgesia devices and the transition from intravenous to oral opioids. The use and doses of intraoperative and postoperative opioids and adjuvant analgesics are recorded on the day of surgery and postoperative day (POD) 1. To allow comparison of opioid doses between the groups, administered opioid doses are converted into morphine equivalents. Also, on POD 7, the use of opioid analgesics after discharge is recorded.

After surgery, the patient is transferred to the PACU (post-anaesthesia care unit). Registration of pain at rest and during movement is performed 2±0.5 hours after arrival at the PACU. Treatment of pain and nausea follows local routines. When local PACU discharge criteria are met, the patients is transferred to the ward where treatment is given according to local routine.

The subject fills in a diary 3 times a day during the first 3 PODs in the hospital or at home, recording pain at rest and during movement, and other indices of recovery. The second QoR-15 is registered by trial staff 24±2 hours after the end of anaesthesia. On POD 7, trial staff call the subject and record the third QoR-15 and obtain information about pain, pruritus, bowel function and postoperative opioid use. If the subject is still hospitalised, data are collected by telephone or on site. Any postoperative complications and length of stay are recorded based on a review of the subject’s record that takes place between POD 31 and 44.

#### Haemodynamics/inflammation subgroup

In a subgroup of 50 patients having surgery at US Linköping, additional haemodynamic measurements will be performed. On arrival at the anaesthesia department, an arterial line is placed and connected to a pulse-contour analysis device (Pulsioflex, Getinge, Gothenburg, Sweden). Mean arterial pressure, cardiac index, stroke volume index, heart rate, stroke volume variation, pulse pressure variation, systemic vascular resistance index, cardiac power index and dPmx (measure of contractility of the heart's left ventricle) are recorded and stored continuously 5 times per min. In addition, the above measurements are recorded manually on a separate sheet on arrival at the operating room, just before anaesthesia, before the start of surgery, 1 hour after the start of surgery, at the end of surgery, on arrival at the PACU and on discharge from the PACU.

**Figure 1:**
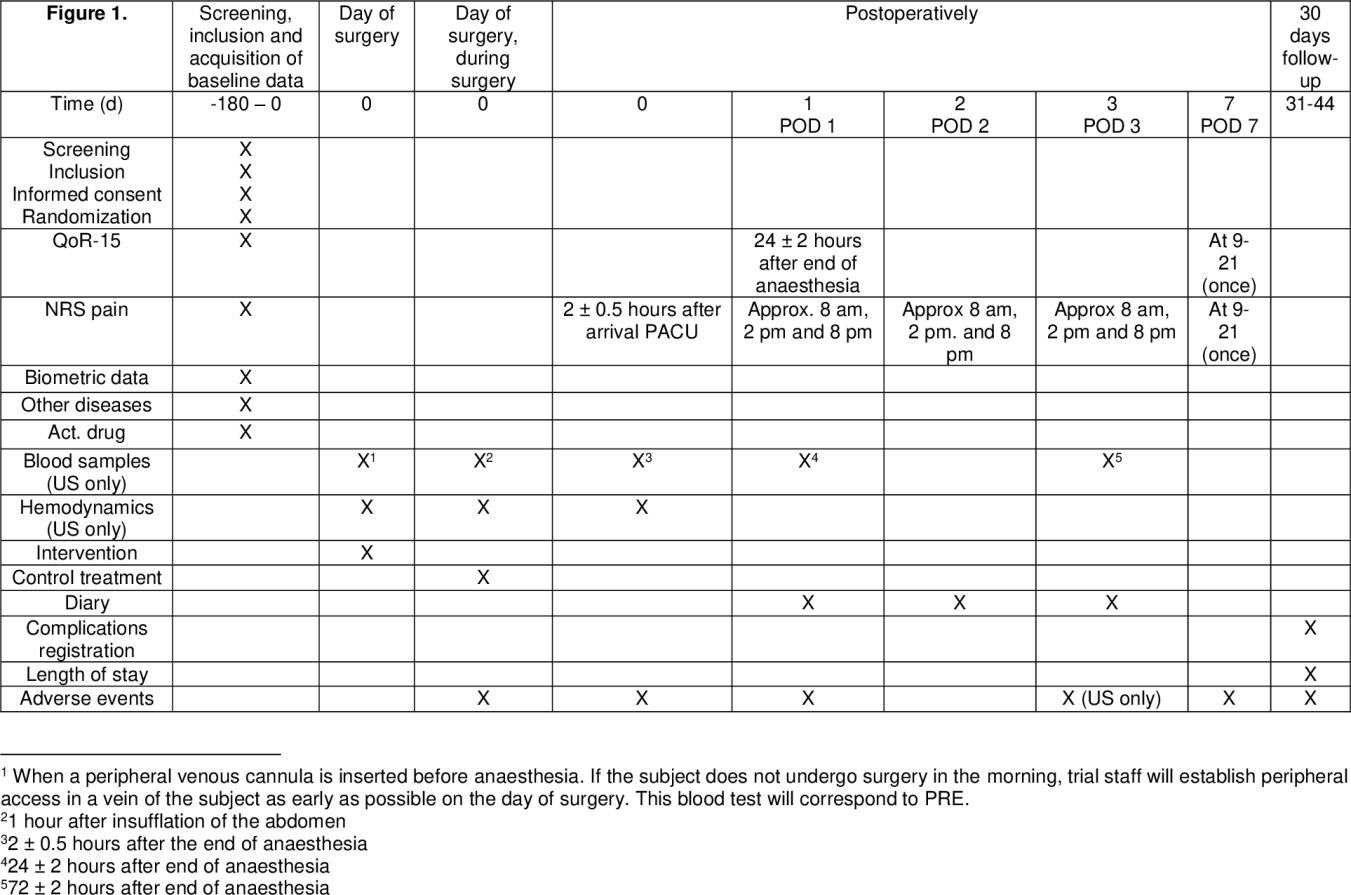
Study flow chart. NRS, Numeric Rating Scale; PACU, post-anaesthesia care unit; POD, postoperative day; PRE, preoperative; QoR, Quality-of-Recovery; US, University Hospital Linköping.

In the same subgroup, blood is sampled for analysing the inflammatory and neuroendocrine response on the day of surgery before arriving in the OR, 1 hour after abdominal insufflation, 2 hours after the end of anaesthesia and on POD 1 and 3 (if not discharged). A study flow chart is shown in figure 1.

### Outcome measures

Primary and secondary outcome measures of this study are shown in table 2 with additional details provided in online supplemental table 1. The primary outcome measure of this study is QoR-15 on POD 1. Our primary hypothesis is that the reduction in QoR-15 from baseline before surgery to POD 1 is at least 8 points less in the morphine spinal group compared with the control group treated with intravenous lidocaine.

**Table 2:** Primary and secondary outcome measures

**1**	**QoR-15 score at POD 1 (primary outcome measure)**
2	QoR-15 score preoperatively
3	QoR-15 score at POD 7
4	Pain (NRS) at rest and during motion 2 hours after arrival to the PACU
5	Pain (NRS) on POD 1–3
6	Pain (NRS) at rest and during motion on POD 7
7	Time from arrival in the OR to start of surgery
8	Time from end of surgery until leaving the OR
9	Incidence of unplanned termination of the lidocaine infusion
10	Amount of remifentanil in patients given remifentanil
11	Amount of intraoperative opioids in patients not receiving remifentanil, expressed in morphine equivalents
12	Length of stay at the PACU/ICU/HDU
13	Amount of opioids administered at the PACU/ICU/HDU during the first 24 hours after end of surgery
14	PONV requiring treatment at 0–6 hours and 6–24 hours postoperatively as well as during the whole postoperative stay
15	‘Time out-of-bed’ on POD 1–3
16	Amount of opioids administered during the first 24 hours at the PACU/ICU/HDU and in the ward
17	First POD passing gases
18	First POD passing stool
19	Incidence of pruritus
20	Length of stay
21	DAOH30
22	Postoperative complications until POD 30
23	Requirement for opioids after discharge
24	Incidence of respiratory depression leading to the use of a μ-antagonist within 48 hours of induction of anaesthesia
25	Intraoperative fluid balance
26	Time with low blood pressure during anaesthesia
27	Lowest MAP within 10 min after induction of anaesthesia
28	Highest MAP within 10 min of start of abdominal insufflation
29	Fraction of patients needing norepinephrine within 15 min after start of abdominal insufflation
30	Fraction of patients needing norepinephrine intraoperatively (later than 15 min after start of abdominal insufflation)
31	Average infusion rate of norepinephrine, in patients receiving norepinephrine, before 15 min after start of abdominal insufflation
32	Average infusion rate of norepinephrine, in patients receiving norepinephrine, after 15 min after start of abdominal insufflation
33	Intraoperative Cardiac Index
34	Intraoperative Stroke Volume Index
35	Intraoperative Cardiac Power Index
36	Intraoperative dPmx
37	Intraoperative Pulse Pressure Variation
38	Intraoperative Stroke Volume Variation
39	Intraoperative dynamic arterial elastance
40	Intraoperative Systemic Vascular Resistance Index
41	Intraoperative heart rate
42	Biochemical markers of inflammation

DAOH30, Days alive and out of hospital at postoperative day 30; dPmx, A measure of contractility of the heart's left ventricle; HDU, high dependency unit; ICU, intensive care unit; MAP, mean arterial pressure; NRS, Numeric Rating Scale; OR, operating room; PACU, post-anaesthesia care unit; POD, postoperative day; PONV, postoperative nausea and vomiting; QoR-15, Quality-of-Recovery 15.

### Safety outcome measures

Safety outcome measures that are recorded in this study are presented in box 1.

Box 1 **Safety outcomes**

- Occurrence of intraoperative or postoperative arrhythmia requiring cardioversion or defibrillation and/or treatment with amiodarone during the stay.
- Occurrence of intraoperative or postoperative hypotension or bradycardia requiring treatment with adrenaline, epinephrine, isoprenaline and/or vasopressin during the stay.
- Occurrence of circulatory instability (arrhythmia or hypotension) postoperatively which prompts a transfer to a higher level of care, for example from the PACU to the ICU or from the ward to the CCU or the ICU during the stay.
- Occurrence of a suspected generalised epileptic seizure intraoperatively or postoperatively during the stay.
- Occurrence of decreased consciousness which causes a move to a higher level of care, for example, from the PACU to the ICU or from the ward to the CCU or ICU, during the stay.
- Presence of opioid-induced respiratory depression that has led to treatment with µ-antagonists and/or invasive respiratory therapy during the stay.
- Occurrence of premature discontinuation of lidocaine infusion.

CCU, coronary care unit; ICU, intensive care unit; PACU, post-anaesthesia care unit.

### Adverse events

Adverse events are defined as incidents, adverse medical events or worsening of an existing medical condition in a subject receiving an investigational drug. Screening for adverse events will be performed at fixed point during the trial (figure 1). In addition, adverse events may be reported at any time during the trial. Adverse events will be assessed for their seriousness and possible relation to the study drugs. Some events are deemed to be naturally related to the surgical procedure and are therefore not registered as adverse events in this trial. These events are specified in box 2.

Box 2 **Events not registered as adverse events in this trial**

- Postoperative pain.
- Postoperative nausea and/or vomiting.
- Constipation.
- Lack of appetite.
- Infectious diseases not fulfilling Clavien-Dindo criteria for grade 2 or above.
- Coagulopathy.
- Pruritus.
- Renal dysfunction not leading to a specific intervention.
- Urinary retention.
- Hypovolaemia and major bleeding related to the surgery.
- Intraoperative or postoperative arrhythmia not requiring cardioversion and/or defibrillation and/or treatment with amiodarone.
- Intraoperative or postoperative hypotension or bradycardia not requiring treatment with epinephrine, isoprenaline and/or vasopressin.
- Transient impact on consciousness in direct connection to the operation that does not lead to a transfer to a higher level of care, for example, from the PACU to the ICU, from the ward unit to the PACU or CCU or ICU.
- Opioid-induced respiratory depression that does not prompt treatment with a µ-antagonist and/or invasive ventilatory therapy during the stay.
- Other known complications clearly related to the intraoperative course such as bleeding (including postoperative), crush injuries, pressure neuropathy, vascular damage and ischaemic damage to the limb or face, including eyes and oral cavity.
- CCU, coronary care unit; ICU, intensive care unit; PACU, post-anaesthesia care unit.

### Statistics

#### Calculation of sample size

In a previous feasibility study including 30 patients undergoing robot-assisted upper urinary tract surgery under general anaesthesia without morphine spinal or intravenous lidocaine, QoR-15 decreased from a median (IQR) preoperative value of 141 (128 – 145) to 106 (88–103) on POD 1 (means (SD) 136 (11) and 105 (22); unpublished data). We assume that variance ((SD)^2^) is 400 in both groups on POD 1. With an MCID of 8, a desired alpha value of <0.05 and a power of 80% 100 subjects are needed in each group (GPower V.3.1.9.7, Heinrich-Heine-Universität Düsseldorf, Germany). To compensate for dropouts, a total of 220 subjects will be included in the trial.

#### Analysis groups

The primary outcome is analysed in the entire trial group according to the intention-to-treat (ITT) principle, except for the subjects who do not undergo the planned robot-assisted surgery within 12 months from inclusion, and/or neither receive intervention nor control treatment, and/or leave the trial and/or die before the POD 1 measurement. The clinical secondary analyses are also analysed in this entire trial group according to ITT. These analyses will be supplemented with:

1. A per protocol (PP) analysis of the primary hypothesis as well as NRS POD 1–3, where subjects who are included in the ITT analysis, however, without sufficient preoperative effect of the spinal analgesia, and/or where lidocaine infusion was prematurely terminated, and/or where the surgery was converted to an open procedure are excluded.
2. An ITT and PP analysis as above where subjects with problematic pain are omitted. Problematic pain is defined here as preoperative opioid use for more than 1 month.

The haemodynamic and biochemical parameters are analysed only in the subgroup that is monitored with advanced haemodynamic monitoring according to the PP principle, that is, subjects with a spinal anaesthetic without adequate preoperative spread, and/or where lidocaine infusion was prematurely terminated, and/or where the surgery was converted to an open procedure are excluded.

Normal distribution will be tested with the Shapiro-Wilks test, except for QoR-15 and NRS data which are assumed to be not normally distributed. All significance tests are done two-sided. A p value lower than 0.05 is considered significant. No corrections for multiple comparisons are made unless otherwise specified.

Single missing values are in principle not imputed. In exceptional cases, if missing data cause an entire data series (in time) to be misleading, the missing data can be imputed with the average value of the two measurements closest in time, provided that this results in a reasonable value.

#### Tests of significance

The primary outcome will be analysed using the Wilcoxon rank sum test (Mann–Whitney U test). For the secondary outcomes, comparisons of single-time values between groups will be analysed using Student’s t-test for normally distributed data, and Wilcoxon rank sum test (Mann–Whitney U test) for not normally distributed data. As mentioned above, QoR-15 and NRS are assumed to be not normally distributed. Proportions will be compared using the χ^2^ test. Longitudinal data such as pain on movement on POD 1 and 3 and perioperative haemodynamics will be compared using mixed effects models.

#### Interim analyses

After 100 and 150 patients have undergone surgery, an interim analysis is done. Recruitment continues during the analyses. The interim analysis examines whether the difference in the primary outcome measure is significant. If this is the case, the trial is stopped and no more patients are included. If this is not the case, the trial continues. The interim analysis is performed by Forum Östergötland and only the above information is provided to the sponsor.

### Blinding

Blinding of the subject and the anaesthesiologist in charge of the patient is not performed. Group allocation is not recorded in the CRF (case record file), only a checkbox with ‘Has the patient received the planned treatment (morphine spinal or lidocaine infusion) as planned yes/no’. The spinal analgesia or lidocaine infusion is documented on the randomisation paper which is kept separately from the CRF. Spinal analgesia or lidocaine infusion is also documented in the usual way in the anaesthesia journal. This information is visible when trial data are transferred from the anaesthesia record to the CRF but not later during data analysis. When the subject is contacted postoperatively, it should not be actively sought whether the subject has received a spinal anaesthetic or not. Should this information still come to light, no note is made of this. However, if the person who collects the QoR-15 on POD 1 has, for some reason, received information about the treatment method before the QoR-15 has been recorded, this must be recorded on the PD (protocol deviation) log. Group affiliation is not noted in the diary. The randomisation code is broken only when data acquisition is completed. In case of emergency, code breaking takes place through a review of the subject’s anaesthesia record.

### Ethics

This study is performed in accordance with the Declaration of Helsinki and Good Clinical Practice. The original study protocol was approved by the Swedish Medical Products Agency on 27 October 2023 (5.1.1-2023-69740). A significant modification was approved on 12 May 2025 (5.1.2-2025-030145). The study is registered at Clinical Trials Information System (CTIS; 2023–5 05 941-21-00) and ClinicalTrials.gov (NCT06349668; date of first registration 29 March 2024). Substantial changes to the signed protocol are only possible through approved protocol changes in the CTIS and will be communicated to all participating sites.

We have shown in a feasibility study that robot-assisted upper urinary tract surgery leads to a significant reduction in early postoperative well-being. Most patients also experience severe pain. There is thus a clear need for strategies that improve well-being after robot-assisted upper urinary tract surgery. The main hypothesis of the trial is that the addition of morphine spinal in robot-assisted upper urinary tract surgery results in improved recovery.

The reduced sympathetic effect resulting from a spinal anaesthetic may result in hypotension, which may require treatment with intravenous fluids and/or vasopressors. This is standard procedure in anaesthesia and is not expected to cause any increased risk to the subjects. In some unusual conditions, such as severe aortic valve stenosis, the risks associated with a reduced sympathetic effect increase and patients with these conditions are excluded.

The risk of severe neurological complications caused by bleeding, trauma or infection in connection with the administration of spinal analgesia has been reported to be approximately 1:25 000.^41^ This should be related to the fact that the risk of death within 30 days after an operation in Sweden has been reported to be 1:50.^42^ Therefore, even a moderate positive effect of morphine spinal on the postoperative course far outweighs its risks. Patients with risk factors for complications related to spinal analgesia such as coagulopathy, previous lumbar spine surgery with osteosynthesis material, severe local or systemic infections and severe spinal deformities are excluded. The application of spinal analgesia will be aborted if it is difficult or if it causes severe discomfort for the patient.

Due to the disadvantages of morphine spinal, it is clinically relevant to investigate whether morphine spinal is actually better than an alternative additional treatment without the disadvantages of morphine spinal. Of the possible strategies considered to have the potential to approach equivalent effect on postoperative recovery as a morphine spinal, only intravenous lidocaine is not associated with renal effects or a negative effect on tumour progression.

Lidocaine given intravenously perioperatively for pain relief is a so-called ‘off-label’ use, similar to many other drugs used in anaesthesia, for example, epidural fentanyl. The use of lidocaine in this way is currently limited in Sweden, but it is routine in many hospitals abroad and is recommended in various international guidelines.^24 25^ The bolus dose and infusion rate in the current trial are well in line with previous studies on the effect of lidocaine as a pain-relieving drug given intravenously.^13^

The lidocaine infusion will be started and finished while the subject is closely monitored in the operating room. Signs of systemic toxicity will thus be detected early and treatment rapidly initiated. The relatively short treatment time means that the risk of accumulation and late toxicity is considered negligible, even in subjects with pre-existing or new-onset renal failure. The infusion is stopped prematurely if severe unexplained hypotension or arrhythmia occurs. Patients with severe kidney or liver failure or advanced heart disease will not be included in the trial. The use of larger doses of local anaesthetic in other ways during the primary operation is not permitted in those receiving lidocaine.

The trial involves the handling of sensitive personal data. The risk of various forms of privacy infringement is minimised through informed consent and pseudonymised processing and storage of research data, including sample material.

### Protocol adherence

Intervention and comparator groups will receive the treatment only on one occasion (preoperatively and intraoperatively, respectively). An anaesthetist will provide the intrathecal analgesia and anaesthesia staff will administer the intravenous lidocaine. Drug administration is routinely documented in the anaesthesia record as well as in a separate trial drug log.

Study staff will visit the subject in the surgical ward to obtain the QoR-15 on POD 1 (primary outcome). Study staff will try to reach the subject on telephone on POD 7 between 09:00 and 21:00. On that occasion, the subject will also be reminded to send in the diary if not already done. For subjects who withdraw from the study, no additional data will be collected; however, previously collected data will be analysed.

### Monitoring

The trial will be monitored by Forum Östergötland before the trial begins, during the course of the trial and after the trial has ended. The first visit at each site will take place after 1–2 subjects have completed the 30-day follow-up in the study. Thereafter, monitoring will take place each time 20 new subjects have completed the 30-day follow-up. The process will be independent of the investigators and the sponsor.

The study also is monitored by a data safety monitoring board, which consists of three individuals with relevant clinical experience. After an introductory meeting, the board and sponsor will meet after the inclusion of the 10th patient participating in the trial. Hereafter, the board will meet at least twice every year.

A safety report is submitted annually to the Swedish Medical Products Agency.

### Ancillary and post-trial care

In case of trial-related adverse events, the subject will be followed by the sponsor in addition to the attending physician until the event has ended or until the condition has become chronic. Regular patient insurance and extended pharmaceutical insurance apply.

### Data protection and storage

Trial data are stored to maintain confidentiality in accordance with national data legislation. Investigator file, consent form, CRF and key to social security number will be stored in locked rooms during the trial. After completion of the study, these data will be archived in accordance with national legislation. All data processed by the sponsor will be pseudonymised and identified by trial ID.

### Dissemination

The results of this study will be presented at national and international meetings and submitted for publication in peer-reviewed international medical journals.

### Trial status

The first patient was included on 9 April 2024. As of 1 October 2025, 54 patients have been included. A second centre has now started recruiting, and a third is expected to follow. Recruitment is expected to be finished by the end of 2027.

## Discussion

In this article, we describe the rationale and design of the SMILe (Spinal Morphine or Intravenous Lidocaine) study, a randomised assessor-blinded multicentre trial, investigating whether morphine spinal is superior to intravenous lidocaine in patients undergoing robot-assisted upper urinary tract surgery under general anaesthesia. Outcome measures include primarily PROM but also measures related to logistics, haemodynamics and inflammation.

At present, these procedures are associated with significant postoperative pain, and additional measures to improve postoperative recovery are thus warranted. Morphine spinal is effective^43^; however, it has disadvantages limiting its use. Other possible methods have side effects related to renal function and/or tumour biology. Lidocaine infusion is a potential tool lacking these side effects; however, its use has been limited in Sweden, probably because of the fear of side effects usually associated with systemic administration of local anaesthetics.

This study will thus provide important information on the use of morphine spinal in robot-assisted procedures, which are expected to increase in frequency. The findings will either confirm the beneficial effects of morphine spinal in these and similar procedures or make it possible to, in good conscience, omit the technique. The study will also provide additional safety data on perioperative lidocaine infusion, which, given its potential favourable role on tumour biology,^44^ might lead to an increasing use of this modality also in other cancer-related surgical procedures.

MCID is defined as the smallest change in a treatment outcome that an individual patient would identify as clinically important and which would justify a change in the patient’s management. Originally, the MCID for the QoR-15 was reported to be 8,^35^ but in a more recent article, the MCID was updated to 6.^36^ Considering the invasive nature of spinal morphine and its related side effects such as hypotension, increased use of resources and the need for increased postoperative monitoring, we chose the more conservative MCID value of 8.

Allowing different types of anaesthetic management can be considered a limitation, as this may introduce confounding effects. After discussion within the trial group, we decided to recommend intravenous anaesthesia; however, inhalational anaesthesia was permitted when preferred because of factors such as TCI(target controlled infusion)-related side effects or local clinical routines. We believe that permitting different anaesthetic regimens enhances external validity, enabling the results to be applied to a broader clinical context.

Strengths of the study include its multicentre design which increases external validity. Also, recovery is measured in multiple ways at multiple time points, as are effects on haemodynamic and inflammatory parameters. The use of an active comparator is a strength since it increases the clinical benefit of key results. The use of a control group (primary or secondary) without additional intervention has been contemplated but rejected for the following reasons:

1. The relevant question is not primarily whether morphine spinal is effective in this setting but whether it is more effective than alternative methods with fewer disadvantages.
2. It is deemed not feasible to perform spinal analgesia blinded. This would require either the administration of morphine spinal after induction of general anaesthesia, which is not a preferred method since it might increase the risk of neurological injury related to the spinal punction, or it would require the omittance of bupivacaine which we feel is an important component of the morphine spinal concept. Therefore, the risk of confounding by placebo expectations is decreased when using an active comparator.
3. It is ethically questionable to assign study patients to a treatment arm which we know is associated with severe postoperative pain.

In conclusion, the SMILe study will establish whether morphine spinal improves postoperative recovery compared with intraoperative lidocaine infusion in patients undergoing robot-assisted upper urinary tract surgery. Also, the effects of these two treatment modalities on haemodynamics and inflammation will be studied.

## Supplementary material

10.1136/bmjopen-2025-113402 online supplemental table 1
