# Supplementary material for: Rationale and design of the SMILe (Spinal Morphine or Intravenous Lidocaine) study: protocol for a multicentre randomised clinical trial in southern Sweden on whether spinal morphine improves postoperative recovery compared to intravenous lidocaine in patients undergoing robot-assisted upper urinary tract surgery
Source: BMJ Open. 2026 May 6;16(5):e113402. doi: 10.1136/bmjopen-2025-113402 (PMC13150920; doi:10.1136/bmjopen-2025-113402)
Supplement: online supplemental table 1 [file bmjopen-16-5-s001.docx]

| Supplementary Table 1. Details on primary and secondary outcomes | | | | |
| --- | --- | --- | --- | --- |
|  | Outcome measure | Analysis metric | Method of aggregation | Time |
| 1 | QoR-15 score at POD 1  (primary outcome measure) | Decrease from preoperative to POD 1 measurement (primary outcome) | Median | First day after surgery |
|  |  | Absolute value | Median |  |
| 2 | QoR-15 score preoperatively | Absolute value | Median | Any time between inclusion and the night before surgery |
| 3 | QoR-15 score at POD 7 | Decrease from preoperative to POD 7 measurement | Median | Seventh day after surgery |
| 4 | Pain (NRS) in rest and during motion 2hrs after arrival to the PACU | Absolute value | Median | 2hrs after arrival to the PACU |
| 5 | Pain (NRS) on POD 1-3 | Absolute value | Median | First, second and third day after surgery |
| 6 | Pain (NRS) in rest and during motion at POD 7 | Absolute value | Median | Seventh day after surgery |
| 7 | Time from arrival in the OR to start of surgery | Absolute value | Median or mean | Time from entering the OR to first incision or start of endoscopy |
| 8 | Time from end of surgery until leaving the OR | Absolute value | Median or mean | Time from end of surgery until leaving  the OR |
| 9 | Incidence of unplanned termination of the lidocaine infusion | Incidence | Proportion | Intraoperatively |
| 10 | Amount of remifentanil in patients given remifentanil | Absolute value | Median or mean | Intraoperatively |
| 11 | Amount of intraoperative opioids in patients not receiving remifentanil, expressed in morphine equivalents | Absolute value | Median or mean | Intraoperatively |
| 12 | Length of stay at the PACU/ICU/HDU | Absolute value | Median or mean | From first to final recording of any vital sign by the electronic patient data  management system, up to 30 days |
| 13 | Amount of opioids administered at the PACU/ICU/HDU during the first 24 hrs after end of surgery | Absolute value | Median or mean | From first to final recording of any vital sign by the electronic patient data  management system, up to 24 hrs after end of surgery |
| 14 | PONV requiring treatment at 0-6 hours and 6-24 hours postoperatively as well as during the whole postoperative stay | Incidence | Proportion | At 0-6 hours and 6-24 hours postoperatively as well as during the whole postoperative stay |
| 15 | ”Time out-of-bed" on POD 1-3 | Absolute value | Median or mean | First, second and third day after surgery |
| 16 | Amount of opioids administered during the first 24 hours at the PACU/ICU/HD and on the ward | Absolute value | Median or mean | During the first 24 hours at the PACU and on the ward |
| 17 | First POD passing gases | Absolute value | Median or mean | From first until seventh day after surgery |
| 18 | First POD passing stool | Absolute value | Median or mean | From first until seventh day after surgery |
| 19 | Incidence of pruritus | Incidence | Proportion | From first until seventh day after surgery |
| 20 | Length of stay | Absolute value | Median or mean | From first until thirtieth day after surgery] |
| 21 | DAOH30 | Absolute value | Median or mean | From first until thirtieth day after surgery |
| 22 | Postoperative complications until POD 30 | Incidence | Proportion | From first until thirtieth day after surgery |
| 23 | Requirement for opioids after discharge | Incidence | Proportion | From first until seventh day after surgery |
| 24 | Incidence of respiratory depression leading to the use of a mu-antagonist within 48 hours of induction of anaesthesia | Incidence | Proportion | From induction of anaesthesia until 48 hours after induction of anaesthesia |
| 25 | Intraoperative fluid balance | Absolute value | Median or mean | Intraoperatively |
| 26 | Time with low blood pressure during anaesthesia | Absolute value | Median or mean and proportion | Intraoperatively |
| 27 | Lowest MAP within 10 minutes after induction of anaesthesia | Absolute value | Median or mean | Within 10 minutes after induction of anaesthesia |
| 28 | Highest MAP within 10 minutes of start of abdominal insufflation | Absolute value | Median or mean | Within 10 minutes of abdominal insufflation |
| 29 | Fraction of patients needing norepinephrine within 15 minutes after start of abdominal insufflation | Incidence | Proportion | From anaesthesia induction until 15 minutes after start of abdominal insufflation |
| 30 | Fraction of patients needing norepinephrine intraoperatively (later than 15 minutes after start of abdominal  insufflation) | Incidence | Proportion | Intraoperatively (later than 15 minutes after start of abdominal insufflation) |
| 31 | Average infusion rate of norepinephrine, in patients receiving norepinephrine, before 15 minutes after start of  abdominal insufflation | Absolute value | Median or mean | From anaesthesia induction until 15 minutes after start of abdominal insufflation until end of anaesthesia,  up to 48 hours |
| 32 | Average infusion rate of norepinephrine, in patients receiving norepinephrine, after 15 minutes after start of abdominal  insufflation | Absolute value | Median or mean | From 15 minutes after start of abdominal insufflation until end of anaesthesia (extubation), up to 48 hrs |
| 33 | Intraoperative Cardiac Index | Absolute value | Median or mean and proportion | Intraoperatively |
| 34 | Intraoperative Stroke Volume Index | Absolute value | Median or mean | Intraoperatively |
| 35 | Intraoperative Cardiac Power Index | Absolute value | Median or mean | Intraoperatively |
| 36 | Intraoperative dPmx | Absolute value | Median or mean | Intraoperatively |
| 37 | Intraoperative Pulse Pressure Variation | Absolute value | Median or mean | Intraoperatively |
| 38 | Intraoperative Stroke Volume Variation | Absolute value | Median or mean | Intraoperatively |
| 39 | Intraoperative dynamic arterial elastance | Absolute value | Median or mean | Intraoperatively |
| 40 | Intraoperative Systemic Vascular Resistance Index | Absolute value | Median or mean | Intraoperatively |
| 41 | Intraoperative heart rate | Absolute value | Median or mean | Intraoperatively |
| 42 | Biochemical markers of inflammation | Absolute value | Median or mean | Day of surgery and first and third day after surgery. |
