# Supplementary material for: Rationale and design of the SMILe (Spinal Morphine or Intravenous Lidocaine) study: protocol for a multicentre randomised clinical trial in southern Sweden on whether spinal morphine improves postoperative recovery compared to intravenous lidocaine in patients undergoing robot-assisted upper urinary tract surgery
Source: BMJ Open. 2026 May 6;16(5):e113402. doi: 10.1136/bmjopen-2025-113402 (PMC13150920; doi:10.1136/bmjopen-2025-113402)
Supplement: Supplementary file 2 [file bmjopen-2025-113402.reviewer_comments.pdf]

PEER REVIEW HISTORY

BMJ Open publishes all reviews undertaken for accepted manuscripts. Reviewers are asked to complete a checklist review form and are provided with free text boxes to elaborate on their assessment. These free text comments are reproduced below.

ARTICLE DETAILS

Title (Provisional)

Rationale and design of the SMILe (Spinal Morphine or Intravenous Lidocaine) study: protocol for a multicentre randomised clinical trial in Southern Sweden on whether spinal morphine improves postoperative recovery compared to intravenous lidocaine in patients undergoing robot-assisted upper urologic tract surgery

Authors

Holmberg, Martin; Chew, Michelle; Nilsson, Lena; Skoglund, Per; Bahlmann, Hans

---

VERSION 1 - REVIEW

**Reviewer**	**1**
**Name**	**Gong, Yuan**
**Affiliation**	**Central People's Hospital of Yichang City, Department of Anesthesiology**
**Date**	**11-Dec-2025**
**COI**	**None**

Well designed protocol.The rationale of the protocol based on the concern of the author's study. The statistic protocol may be suited for the protocol.

**Reviewer**	**2**
**Name**	**Beaussier, Marc**
**Affiliation**	**Institut Mutualiste Montsouris, Anesthesiology**
**Date**	**22-Dec-2025**
**COI**	**None**

This manuscript describes a study protocol comparing spinal morphine (+ bupivacaine) and intravenous lidocaine for pain relief and recovery course after robotic upper urologic surgery. 220 patients were planned to be included. The investigation started on April 2024 and is expected to be completed on 2027.

The subject is undoubtedly highly pertinent and meaningful. The place of morphine spinal analgesia in this setting is still under controversy and the present approach would be able to positively fuel the debate. One of the major interests of this study is to focus on patient's experience by using the QoR 15 score. Furthermore, besides to pain relief, parameters related to recovery course and rehabilitation are extensively screened. In contrast to the statement p6; the absence of control group with no treatment cannot be considered as a limitation. The infusion of intravenous lidocaine in the control group is rather an appropriate comparator at the light of recent knowledge in the topic.

The manuscript is well written and easy understandable. However, several points could nevertheless be modified to still improve the information delivered.

- Information about analgesic strategy, aside from the technics under comparison, would deserve to be described in more details. P15; because opiate consumption was a criteria of comparison, it is absolutely necessary to provide additional data on how morphine was administered. Does patient-controlled morphine analgesia be used? At which time the pump was initiated? Were there any co-analgesics (NSAIDs …) what about transition to oral administration? Does local infiltration of trocar ports be applied?

- Intraoperative anesthetic management is not uniform (inhalational vs intravenous). This could introduce some difference on immediate postoperative outcomes (PONV, residual sedation…). Isn't it a limitation?

- P16; Criteria of intraoperative remifentanil administration should be provided to make any between-group comparison valid.

- Spinal morphine was already evaluated with a specific emphasize on postoperative recovery course (after open colorectal surgery). See the paper by Beaussier M et al. RAPM 2006 that should be quoted.

- Sample size calculation was built on a 8-points difference on QoR15, assuming the same variability after surgery in both groups (p21 line 18-23). This should be explained more precisely. Because of the mention about a feasibility study that has been done, some details should be provided. On which criteria these expected differences are related? is it on pain intensity ? A short paragraph on this point should be added in the discussion section.

- MCID is not defined in abbreviation table. P23 line 23-26; the sentence "considering…" is not clear. Please explain what is the relationship between the invasive nature of spinal morphine and the MCID ?

VERSION 1 - AUTHOR RESPONSE

- Reviewer: 1
Well designed protocol.The rationale of the protocol based on the concern of the author's study.The statistic protocol may be suited for the protocol.

Authors' reply: Thank you for reviewing our work and your positive feedback.

Reviewer: 2
The manuscript is well written and easy understandable. However, several points could nevertheless be modified to still improve the information delivered.
- Information about analgesic strategy, aside from the technics under comparison, would deserve to be described in more details. P15; because opiate consumption was a criteria of comparison, it is absolutely necessary to provide additional data on how morphine was administered. Does patient-controlled morphine analgesia be used? At which time the pump was initiated? Were there any co-analgesics (NSAIDs …) what about transition to oral administration? Does local infiltration of trocar ports be applied?

Authors' reply: We agree that this is important information, and it is now added to the section "Intervention and measurements" in the revised version as follows:

"Additional intraoperative use of local anaesthetics, e.g. for infiltration of trocar ports, is not allowed, with the exception of wound infiltration in case of unplanned conversion to laparotomy in patients in the morphine spinal group. Paracetamol 1g iv or po is given every sixth hour to all patients. The addition of other adjuvant analgesics such as NSAIDs, gabapentin, COX-2 inhibitors, clonidine and magnesium, is not permitted intraoperatively. Postoperatively, opiates and adjuvant analgesics are administered at the discretion of the responsible nurse or physician according to local routines. This includes the use of patient-controlled analgesia devices and the transition from intravenous to oral opioids. The use and doses of intra- and postoperative opioids and adjuvant analgesics are recorded on the day of surgery and POD 1. To allow comparison of opioid doses between the groups, administered opioid doses are converted into morphine equivalents. Also, at POD 7 the use of opioid analgesics after discharge is recorded."

The postoperative use of patient-controlled analgesia (PCA) and the transition to oral medication are not specified in the original protocol. PCA is usually not applied in these patients but might be required in case of unplanned conversion to laparotomy. The transition to oral medication follows local ward routines. Doses of opioid administered via these and other routes are recorded on the day of surgery and POD1. We will include a note on the use of PCA and the transition to oral medication in a subsequent revision of the original protocol as registered at the EMA.

- Intraoperative anesthetic management is not uniform (inhalational vs intravenous). This could introduce some difference on immediate postoperative outcomes (PONV, residual sedation…). Isn't it a limitation?

Authors' reply:
Thank you for this comment regarding the issue of accepting different anaesthesia regimens. We agree that this could have a confounding effect and thus could be regarded as a limitation. In the study, intravenous anaesthesia (TCI) is recommended, however inhalational anaesthesia is allowed should this be preferred because of e.g. TCI related side-effects or local routine. We also argue that allowing different anaesthetic regimens improves external validity.

We have added the following in the "Discussion section" to clarify this issue:
"Allowing different types of anaesthetic management can be considered a limitation, as this

may introduce confounding effects. After discussion within the trial group, we decided to recommend intravenous anaesthesia; however, inhalational anaesthesia was permitted when preferred because of factors such as TCI-related side effects or local clinical routines. We believe that permitting different anaesthetic regimens enhances external validity, enabling the results to be applied to a broader clinical context."

- P16; Criteria of intraoperative remifentanil administration should be provided to make any between-group comparison valid.

Author's reply: We refer to the reply above to the previous two questions by the reviewer and the modifications to the manuscript based on them. As mentioned most patients will receive a propofol-remifentanil based anaesthesia but variations are allowed. The balance between propofol and remifentanil infusions is guided by BIS. All administered doses of propofol, remifentanil and other opioids and intravenous anaesthetic agents are recorded.

- Spinal morphine was already evaluated with a specific emphasize on postoperative recovery course (after open colorectal surgery). See the paper by Beaussier M et al. RAPM 2006 that should be quoted.

Author's reply: We are grateful for the reviewer notifying us about this study. As suggested we reference it now in the Discussion section as an example of the effectiveness of spinal morphine.

- Sample size calculation was built on a 8-points difference on QoR15, assuming the same variability after surgery in both groups (p21 line 18-23). This should be explained more precisely. Because of the mention about a feasibility study that has been done, some details should be provided. On which criteria these expected differences are related? is it on pain intensity ? A short paragraph on this point should be added in the discussion section.

Authors' reply: We agree that more information needs to be provided about the feasibility study and the sample size calculation and have rephrased this passage as follows:

"In a previous feasibility study, including 30 patients undergoing robot-assisted upper urologic tract surgery under general anaesthesia without morphine spinal or intravenous lidocaine, QoR-15 decreased from a median (IQR) preoperative value of 141 (128 – 145) to 106 (88 – 103) on POD 1 (means (SD) 136 (11) and 105 (22)) (Holmberg et al., submitted). We assume that variance ((standard deviation)2) is 400 in both groups on POD 1. With an MCID of 8, a desired alpha value of < 0.05 and a power of 80% 100 subjects are needed in each group (GPower 3.1.9.7)."

Because our previous feasibility study (Holmberg et al.) is currently under review, detailed information on the specific QoR-15 items with declining scores cannot be presented at this stage. However, we expect improvements in the domains of rest, well-being and pain.

- MCID is not defined in abbreviation table.

Authors' reply: Thank you for this remark. We have corrected this and have also made a few stylistic corrections to the list of abbreviations.

- P23 line 23-26; the sentence "considering…" is not clear. Please explain

what is the relationship between the invasive nature of spinal morphine and the MCID ?

Authors´reply: Thank you for letting us clarify the relationship between the invasive nature of spinal morphine and the MCID. Originally the MCID for the QoR-15 was reported to be 8 [ref Myles 2016] but in a more recent article the MCID was updated to 6 [ref Myles 2021]. Considering the invasive nature of morphine spinal and its related side effects such as hypotension, increased use of resources and the need for increased postoperative monitoring, we chose the more conservative MCID value of 8.”

We have added the following passage in the section “Discussion”:

“Minimal clinically important difference (MCID) is defined as the smallest change in a treatment outcome that an individual patient would identify as clinically important and which would justify a change in the patient's management. Originally the MCID for the QoR-15 was reported to be 8 [35] but in a more recent article the MCID was updated to 6 [36]. Considering the invasive nature of morphine spinal and its related side effects such as hypotension, increased use of resources and the need for increased postoperative monitoring, we chose the more conservative MCID value of 8.”

Thank you for reviewing our work.

VERSION 2 - REVIEW

**Reviewer**	**2**
**Name**	**Beaussier, Marc**
**Affiliation**	**Institut Mutualiste Montsouris, Anesthesiology**
**Date**	**09-Apr-2026**
**COI**	

The authors have done a good correction. Every items required by the reviewer have been answered iappropriately.
